# Artificial Intelligence in Advancing Algal Bioactive Ingredients: Production, Characterization, and Application

**DOI:** 10.3390/foods14101783

**Published:** 2025-05-17

**Authors:** Bingbing Guo, Xingyu Lu, Xiaoyu Jiang, Xiao-Li Shen, Zihao Wei, Yifeng Zhang

**Affiliations:** 1 College of Chemistry and Life Science, Beijing University of Technology, Beijing 100124, China; guobingbing@bjut.edu.cn (B.G.);; 2 School of Public Health, Zunyi Medical University, Zunyi 563000, China; xiaolishen1983@163.com; 3 College of Food Science and Engineering, Ocean University of China, Qingdao 266404, China; weizihao@ouc.edu.cn; 4 Department of Food Safety and Health, School of Advanced Agricultural Sciences, Peking University, Beijing 100871, China

**Keywords:** artificial intelligence, algal bioactive compounds, AI-based data analysis, AI-based imaging, machine learning

## Abstract

Microalgae are capable of synthesizing a diverse range of biologically active compounds, including omega-3 fatty acids, carotenoids, proteins, and polysaccharides, which demonstrate significant value in the fields of functional foods, innovative pharmaceuticals and high-value cosmetics. With advancements in biotechnology and the increasing demand for natural products, studies on the functional components of algae have made significant strides. However, the commercial utilization of algal bioactives still faces challenges, such as low cultivation efficiency, limited component identification, and insufficient health evaluation. Artificial intelligence (AI) has recently emerged as a transformative tool to overcome these technological barriers in the production, characterization, and application of algal bioactive ingredients. This review examines the multidimensional mechanisms by which AI enables and optimizes these processes: (1) AI-powered predictive models, integrated with machine learning algorithms (MLAs), Industry 4.0, and other advanced digital systems, support real-time monitoring and control of intelligent bioreactors, allowing for accurate forecasting of cultivation yields and market demand. (2) AI facilitates in-depth analysis of gene regulatory networks and key metabolic pathways, enabling precise control over the biosynthesis of targeted compounds. (3) AI-based spectral imaging and image recognition techniques enable rapid and reliable identification, classification, and quality assessment of active components. (4) AI accelerates the transition from mass production to the development of personalized medical and functional nutritional products. Collectively, AI demonstrates immense potential in enhancing the yield, refining the characterization, and expanding the application scope of algal bioactives, unlocking new opportunities across multiple high-value industries.

## 1. Introduction

Algae are a diverse group of aquatic organisms that primarily convert energy through photosynthesis, encompassing a wide range of species from microscopic unicellular microalgae to large multicellular seaweeds. Rich in functional compounds, algae hold significant promise for various applications in health foods, pharmaceuticals, and high-value cosmetics, exemplified by products such as algal DHA oil and seaweed powder. Algae also contain unique bioactive components, such as fucoxanthin, which offer specialized functional benefits [1]. As research and technology continue to advance, the potential for algae utilization is expanding, including the extraction of compounds like astaxanthin from red algae, which serves as a valuable functional ingredient. The bioactive components derived from algae are increasingly applied in pharmaceuticals and cosmetics. For instance, β-carotene is recognized for its tumor-inhibiting properties and ability to boost white blood cell counts [2], while algal polysaccharides exhibit notable antitumor activity [3]; carotenoids and astaxanthin are celebrated for their potent antioxidant effects [4]. Moreover, algae are characterized by a short growth cycle, high production efficiency, and the ability to thrive without occupying arable land traditionally used for food crops [5]. As such, the maximization of algae’s functional components is critical for the development of sustainable food and medicinal products, playing an essential role in global health and wellness.

However, conventional methods for producing functional compounds from algae—including autotrophic or heterotrophic cultivation, biomass harvesting, and product extraction—are often hindered by low yields, high costs, and limited economic viability. These challenges have resulted in unsatisfactory industrial scalability and have significantly restricted the commercialization and broader application of algal-derived products. For instance, the production of astaxanthin from naturally occurring red algae requires the construction of extensive piping systems or large-scale culture ponds, along with precise light treatments [6]. These processes are resource-intensive in terms of labor, materials, and capital investment and are further constrained by geographical and climatic conditions. Additionally, the astaxanthin content in natural red algae must be extracted prior to quantification, leading to potential issues such as batch inconsistency and unstable yields. Also, ensuring a sustainable supply of omega-3 fatty acids and the isolation of EPA and DHA from algae are the biggest challenges in the area [7,8]. Moreover, the safety and functionality of compounds like astaxanthin—and other newly discovered bioactive ingredients—must be rigorously validated through long-term biochemical assays, animal studies, and human trials. Consequently, further study and technological innovation are essential to advance the development and sustainable utilization of algal resources.

With respect to addressing the production challenges associated with algal bioactive compounds, the integration of AI-driven intelligent systems and dynamic models offers promising solutions. These technologies can significantly enhance the efficiency of algal cultivation and processing systems, thereby unlocking the full potential of algal-derived functional ingredients for broader applications in pharmaceuticals, food, and agriculture. In the production of algal active ingredients, AI technologies have already demonstrated the ability to improve both yield and product quality [9,10,11]. For example, AI-based regulatory systems utilizing transfer learning can dynamically adjust photobioreactor parameters in real time, autonomously generating optimized control strategies tailored to specific algal species. This approach effectively overcomes the lag associated with traditional regulatory methods [12,13]. Moreover, Industry 4.0 frameworks—incorporating AI and machine learning (ML) models—enable real-time monitoring and optimization of algal cultivation processes. These systems continuously update operational parameters to maintain optimal conditions, predict biomass yields, and forecast future production demands [14], thereby supporting the precise quantification of bioactive ingredient outputs. In addition, AI combined with ML algorithms can be applied to analyze transcriptomic and proteomic data, allowing researchers to uncover novel gene–pathway relationships and regulatory mechanisms. This insight facilitates the identification and validation of optimal genetic pathways in stable microalgae strains, significantly accelerating biomass accumulation and improving overall system efficiency [10]. An artificial neural network (ANN) combined with a genetic algorithm (GA) improved the algal biomass productivity by about 57% [15]. For characterization, intelligent systems and dynamic AI models leverage advanced techniques such as image recognition and spectral analysis to identify and assess algal functional components [14,16,17]. Spectral remote sensing, which integrates imaging and spectroscopy, can detect subtle spectral variations among different algal species, enabling precise species identification and component characterization [16,17]. Notably, the use of Convolutional Neural Networks (CNNs) in conjunction with hyperspectral imaging and portable diagnostic devices allows for rapid extraction of morphological and pigment-related features. This innovation supports accurate classification at the subspecies level, overcoming challenges posed by structural heterogeneity and enhancing the spatial resolution of component distribution within complex biological matrices. Furthermore, AI-driven ML approaches are reshaping the application landscape of algal bioactives—from bulk production toward the development of personalized medical and nutritional products. This shift expands the potential of algae-derived compounds in high-value sectors such as cosmetic dermatology and biopharmaceuticals. In functional food engineering, for instance, AI has enabled the transformation of photosensitive algal metabolites into intelligent sensing tools [14], opening new application scenarios in areas like real-time monitoring of food freshness.

This review summarizes intelligent technologies used in accelerating algal production systems, including those facilitating real-time monitoring of culture processes and gene regulation. Also, AI-driven image technologies and spectral analysis technology would improve accuracy in characterizing algal bioactive compounds. Moreover, AI stimulates innovative applications of algal bioactive compounds from pharmaceuticals, as well as foods. Finally, we discuss the technological challenges and future directions of AI utilization in relation to algal bioactives. These insights will serve as a valuable reference for researchers and practitioners seeking to further innovate within the algal biotechnology field.

## 2. AI in Advancing Production of Algal Bioactive Ingredients

**Figure 1:**
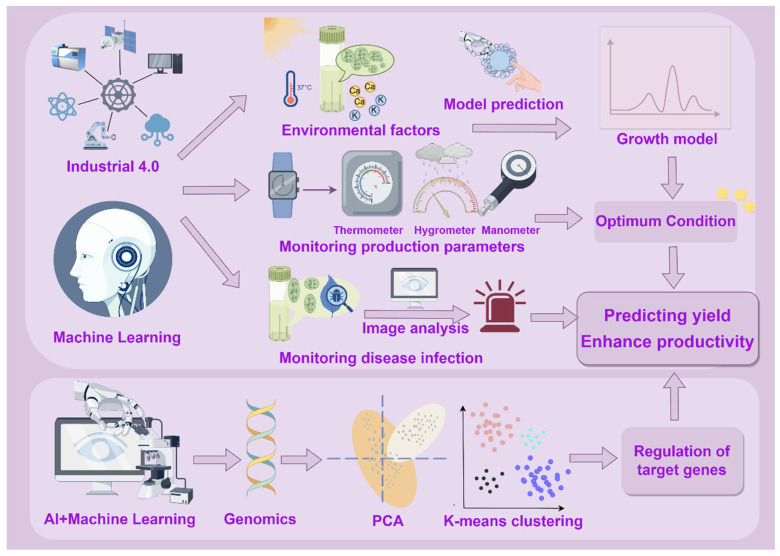
AI-integrated technologies in the production of algal bioactive compounds.

Through the optimization of metabolic engineering and the precise regulation of the cultivation process, AI has emerged as a powerful tool for the efficient production of algal bioactive compounds, and multidimensional experimental validations have underscored its potential for industrial-scale applications (Figure 1). AI enhances the yield and quality of algal bioactives through two key mechanisms: (1) The first key mechanism is real-time monitoring and predictive control. AI systems enable continuous monitoring of algal culture conditions, allowing for accurate prediction of future production trends and market demand [14,16,17]. These systems can also provide early warnings in response to operational anomalies, minimizing the risk of production loss or quality deviation. (2) The second key mechanism is the optimization of gene regulatory networks. AI and machine learning algorithms (MLA) can model and predict complex interactions within genetic and metabolic pathways. This capability enables the fine-tuning of gene regulatory mechanisms in microalgae, thereby significantly improving biomass accumulation and enhancing the overall efficiency of bioactive compound synthesis [10].

### 2.1. AI Enables Real-Time Monitoring of Algae Growth and Prediction of Algal Bioactive Ingredient Yields

The growth of algae and the synthesis of functional algal components are influenced by a variety of environmental factors, including light intensity, temperature, pH, and nutrient availability [18]. AI-enabled intelligent systems and dynamic models (Industry 4.0, ML, MLAs) can analyze the relationship between these factors and microalgae growth to find the optimal cultivation conditions for each microalgae species so as to build accurate growth models, predict the growth trends of algae under different environmental conditions, and automatically adjust the cultivation conditions for accurate feeding to improve growth rate and yield [9,10]. In addition, AI-driven real-time monitoring technology can also achieve early warning and control of diseases. For instance, through image recognition technology and deep learning algorithms (DLAs), AI can quickly identify abnormalities in the process of algae cultivation, such as disease infection, malnutrition, etc., and provide early warnings and carry out corresponding interventions to ensure the healthy growth of algae [9,19]. Currently, AI-driven real-time monitoring to predict and make decisions about the future demand and production of algal cultures involves three main modeling systems, namely Industry 4.0 technology, AI-ML, and AI-MLA models.

#### 2.1.1. AI and MLAs Assist Industry 4.0 Technology for Adaptive Process Control

Industry 4.0 integrates automation, sensors, and ML to create adaptive manufacturing systems capable of dynamically adjusting production processes based on real-time data. Its technical architecture comprises three levels, including (1) data collection—Internet of Things (IoT) technology enables real-time communication and data exchange between devices; (2) data processing—cloud computing provides elastic computing resources to support real-time analysis, while big data technology achieves data-driven decision-making through process optimization and demand forecasting; and (3) intelligent application—AI and ML, combined with the virtual reality interaction capability of cyber-physical systems (CPSs), build dynamic digital models of physical processes through digital twin technology. This technology creates a closed-loop control system in the production of algal active ingredients. Firstly, a plug-and-play IoT sensor network allows operators to monitor algal culture environmental parameters and productivity in real time [20]. Secondly, AI models, such as CNNs and artificial neural network (ANNs), analyze historical data to model the nonlinear relationships between culture conditions and the yield of rockweed xanthophylls, enabling autonomous prediction of optimal extraction conditions [19,21]. At the same time, AI systems based on computer vision can complete the online quantification of metabolites through image color space analysis and in combination with regression statistics [9]. Ultimately, the digital twin system integrates all sensor data to construct a dynamic mirror of the cultivation process in virtual space, predicting the output trend of algal functional components in advance through simulation. This allows for dynamic adjustments to actual production parameters to meet expected product demand and minimize waste.

Integrating AI with MLA enhances real-time monitoring and optimizes predictive models by improving data processing efficiency in the production of algal active ingredients. By connecting various environmental sensing devices, an AI-MLA system can simultaneously track key indicators such as temperature and humidity during algal cultivation. This capability enables real-time detection of equipment anomalies and prediction of product quality fluctuations through trend analysis. Specifically, as the system continuously acquires production environment parameters, advanced algorithms process large datasets instantly. By identifying complex correlations within the data, the system can accurately determine optimal cultivation conditions to increase yields of algal active ingredients. Furthermore, integrating AI-MLA with various sensors and monitoring devices facilitates multifunctional real-time monitoring. For example, combining AI-MLA with temperature, humidity, and pressure sensors allows for real-time monitoring of multiple parameters in the production environment. Analyzing these data enables the prediction of equipment maintenance needs and potential product quality issues [11]. This efficient data processing capability allows AI-MLA to respond swiftly to changes in real-time monitoring forecasts and to learn and capture nonlinear patterns and relationships from large datasets. Consequently, it can predict optimal conditions that significantly affect the extraction yields of microalgae bioactive compounds, leading to better optimization of microalgae culture systems and more accurate forecasting results [14,22].

#### 2.1.2. AI and ML Facilitate Prediction of Algal Bioactive Ingredient Productivity

ML serves as a robust system for knowledge acquisition and integration, employing inductive reasoning to generalize relationships between inputs and outputs, thereby guiding decision-making in novel environments. In practical applications, optimized ML models leverage high-resolution microscopy or remote sensing imagery to acquire algal samples images. Subsequently, deep learning (DL) models, particularly CNNs, automatically extract morphological features of algae, such as cell shape, size, and spatial arrangement. These extracted features are then fed into pre-trained classification models and matched with a pre-determined algae database, ultimately resulting in precise algal identification and classification. In addition, the developed models efficiently process large-scale image datasets, making them highly suitable for algae monitoring and classification tasks across diverse spatial scales [14]. Coşgun et al. initially investigated the effects of various influencing factors, including algal species, culture conditions, reactor type, nutrient concentration, stress conditions, cell disruption mode, light intensity, amount of CO_2_, and lipid extraction solvent, on biomass and lipid production by using ML predictive modeling. The research team incorporated the nonlinear relationship between algal physiological properties, bioreactor fluid dynamics, nutrient restriction strategies, and secondary metabolite synthesis pathways into a unified modeling framework, thus achieving a comprehensive optimization of the yield of microalgae active ingredient production and lipid metabolism pathways [12].

Particularly, Random Forest (RF), as an integrated ML method, demonstrates significant advantages in accurately predicting algal active ingredient production by constructing and combining multiple decision trees. The strength of this approach lies in generating numerous independent decision trees through random sampling and feature selection, effectively minimizing overfitting and enhancing model generalization. In the prediction of algal active ingredient production, RF efficiently captures complex nonlinear relationships and identifies the influence of key environmental factors—such as light intensity, temperature, and nutrient concentration—as well as their interactions affecting algal growth and metabolite accumulation. Furthermore, by evaluating feature importance, RF can highlight the most influential variables driving algal productivity, providing critical insights for optimizing culture conditions and improving overall production efficiency. With its high predictive accuracy, RF represents a powerful analytical tool within algal biotechnology, facilitating large-scale production and supporting applied research of algal active ingredients [10,11].

Furthermore, the ML framework adopts cross-validation to minimize prediction errors by converting intricate temporal characteristics of algal metabolism into quantifiable parameters. Studies have demonstrated that specific environmental stresses, such as sulfur (S) stress, can induce a significant redistribution of metabolic fluxes. In particular, when the carbon-to-nitrogen ratio within the algal culture environment exceeds a critical threshold, triacylglycerol synthesis rates markedly increase [23]. These metabolic insights form the foundational principles for developing intelligent regulation systems. By continually monitoring key culture parameters, including pH and dissolved oxygen levels, these systems can proactively predict algal growth states and dynamically adjust culture conditions to optimize performance [12,14].

### 2.2. AI Optimizes the Gene Regulation Mechanism in Algal Bioactive Compound Production by Predicting Gene–Pathway Network Associations

To address current challenges in algal active ingredient research and to advance sustainable food production and the circular economy, the integration of AI with mathematical modeling emerges as a promising strategy for processing and analyzing algal data. This combined approach can optimize the gene regulatory mechanisms for the efficient production of bioactive components. By accurately predicting associations between genes and metabolic pathways, AI facilitates enhancements in algal growth and protein synthesis and improvements in the flavor and organoleptic properties of bioactive ingredients [10]. Furthermore, metabolic flux analysis (MFA) integrated with AI can identify alternative biosynthetic pathways for critical amino acids, such as isoleucine, ultimately enhancing carbon fixation efficiency and biomass production. AI can similarly aid in elucidating pathways that enhance astaxanthin synthesis and regulate hydrogen production by modulating hydrogenase activity [10].

Further, AI-ML can analyze comprehensive transcriptomic and proteomic datasets from numerous microalgae studies, thereby predicting intricate gene–pathway network interactions and uncovering previously unknown regulatory mechanisms [10]. Techniques such as principal component analysis (PCA), RF, and k-means clustering facilitate the prediction and ranking of critical metabolic pathways based on gene expression levels and statistical validation. This predictive ranking enables experimental verification and optimization of metabolic pathways in stable microalgal strains, significantly expanding the applicability of algal bioactives within the food industry [10]. Currently, extensive algal genomic and transcriptomic resources are available, including over 6350 algal gene expression datasets in the Gene Expression Omnibus database and 230 algal genome sequencing projects listed in the Genome Online Database (GOLD) and PhycoCosm at EMBL-EBI. Utilizing these well-annotated genomic resources, AI-driven transcriptomics can provide profound insights into critical metabolic pathways, their enzymes, and regulatory elements, including transcription factors and promoters. Consequently, this facilitates the experimental validation and optimization of targeted gene expression pathways in robust microalgal strains [10]. Based on advanced data mining and analysis, AI technologies enable the prediction of associations between genes and pathway networks to optimize gene regulatory mechanisms in microalgal production, which represents a significant breakthrough in algal biotechnology.

## 3. AI Facilitates the Characterization of the Bioactive Ingredients of Algae

**Figure 2:**
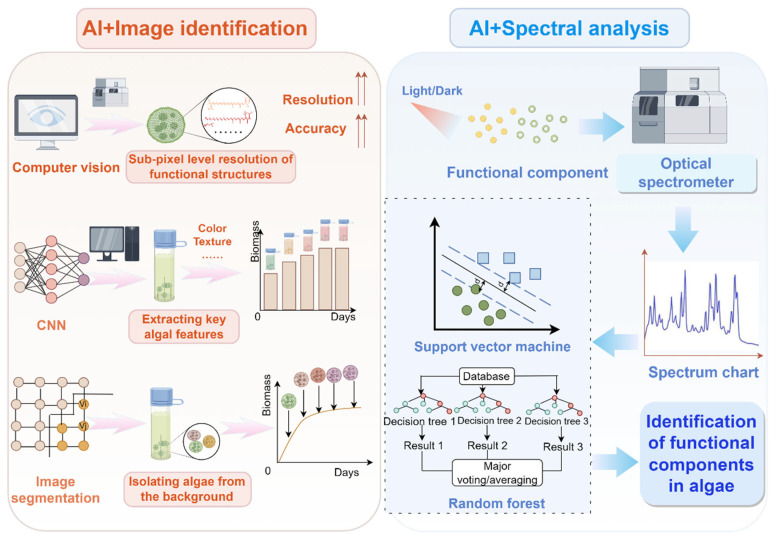
AI-driven technologies in the characterization of algal bioactive compounds. The upper arrows indicate “increase” or “enhance”. The circles indicate algae characters extracted from the culture system. The dotted lines indicate ranges from the central line.

The bioactive components of algae mainly include polysaccharides, proteins, carotenoids, polyphenols, sterols, and terpenes, each of which have distinct bioactive properties. Polysaccharides, such as paramylon, have anti-obesity and anti-inflammation activities [24]; proteins including phycobilin and algal lectin demonstrate abilities to lower blood pressure and regulate blood lipid levels [25]; carotenoids, such as astaxanthin and fucoxanthin, possess antioxidant and weight management effects [26,27]; terpenoids exhibit bactericidal, antibacterial, and antitumor properties [28]; polyphenols provide antioxidant and antibacterial benefits [29]; and sterols are known to regulate hormonal balance and reduce cholesterol levels [30]. However, the accurate characterization of these bioactive compounds faces significant challenges due to structural heterogeneity, dynamic metabolism, and detection bottlenecks, especially for components present in low abundance. AI, together with image identification and spectral analysis, has paved the way for the characterization of algal bioactive compounds (Figure 2).

### 3.1. AI Combined with Image Recognition Technology Overcomes Limitations in Spatial Distribution Analysis of Bioactive Components

The integration of AI with unmanned aerial vehicle (UAV) remote sensing technology and advanced AI algorithms facilitates intelligent, automated, and real-time identification and quantification of algae through a structured workflow involving data collection, image preprocessing, feature extraction, model training, and evaluation. Specifically, AI can leverage CNNs, computer vision, and transfer learning methodologies (e.g., ResNet, VGG) to quantify bioactive compounds, such as astaxanthin, by analyzing color shifts in algal cultures [6]. CNN-based AI frameworks automatically extract critical image features, including color and texture, and establish nonlinear correlations between observed color variations and bioactive compound yields through DL [21]. Image segmentation techniques, notably semantic segmentation, precisely isolate algae from complex backgrounds, enhancing data quality for subsequent yield prediction [31,32]. Additionally, converting color spaces (e.g., RGB to HSV or Lab) significantly amplifies subtle color differences, improving analytical precision [33]. Feature extraction approaches like color histograms and texture analysis combined with MLA identify key yield-correlated features. Simultaneously, time-series analyses using Long Short-Term Memory (LSTM) networks capture dynamic color changes, enabling accurate temporal yield predictions. Data augmentation techniques, including rotation, scaling, and flipping, further enhance model accuracy and generalizability [34]. Leveraging transfer learning through models pre-trained on extensive datasets, such as ResNet and VGG, achieves rapid convergence and robust prediction accuracy [35]. Multispectral and hyperspectral imaging extend the spectral dimensionality of algae analysis, capturing reflectance properties across diverse wavelength bands to strengthen yield predictions [13].

Remarkably, CNN architectures integrated with attention mechanisms effectively capture morphological features of algal cellular structures like cell walls and thylakoids, significantly enhancing the localization and identification of bioactive components, including carotenoid particles and Zietol storage vesicles [36]. For example, intelligent sensing systems combining computer vision and multispectral imaging can achieve sub-pixel resolution in monitoring molecular conformational changes of carotenoids like astaxanthin. Such non-destructive monitoring methods allow for precise extraction of characteristic spectral data for intermediate compounds using CNNs, complemented by LSTM-based dynamic recursive modeling to surpass traditional colorimetric methods [17,21].

### 3.2. AI-Supported CNNs and Spectral Analysis Technology Address Structural Heterogeneity and Improve Accuracy in Characterizing Algal Bioactive Compounds

The synergistic integration of AI and spectral analysis technologies introduces a novel methodological paradigm in algal bioactive research, significantly enhancing species identification accuracy, physiological state assessment, biomass prediction, and resolution of structural heterogeneity issues. Spectral analysis exploits the unique light absorption properties of specific algal pigments, capturing multidimensional spectral information in ultraviolet, visible, and near-infrared wavelengths related to algal pigment composition, cellular structure, and metabolic activities [37]. Utilizing sophisticated data processing capabilities, AI combined with ML and DLA efficiently processes high-dimensional spectral datasets. Supervised learning algorithms, including support vector machines and RFs, classify spectral data based on pigment-specific absorption peaks (e.g., chlorophyll a, chlorophyll b, carotenoids, phycocyanin), facilitating rapid species identification [38]. CNN-based DL models extract multi-layered features directly from raw spectral inputs through end-to-end learning, significantly outperforming traditional manual feature extraction methods and enhancing classification accuracy [17,21].

To address structural heterogeneity in algal bioactive characterization, AI-integrated spectral analysis efficiently deconvolves overlapping spectral peaks and precisely distinguishes isomeric features. Hierarchical CNN architectures combined with self-attention mechanisms extract multidimensional frequency-domain characteristics from infrared and Raman spectral data, enhancing sensitivity to subtle vibrational mode differences among isomers [17]. The introduction of generative antagonistic networks simulates chemical shift perturbations to effectively resolve overlapping spectral signals caused by homologous functional group similarities [14], which significantly improves the accuracy of identifying cis- and trans-isomers among bioactive compounds such as ziziol homologues and carotenoids.

## 4. AI Expands the Application of the Functional Ingredients of Algae

**Figure 3:**
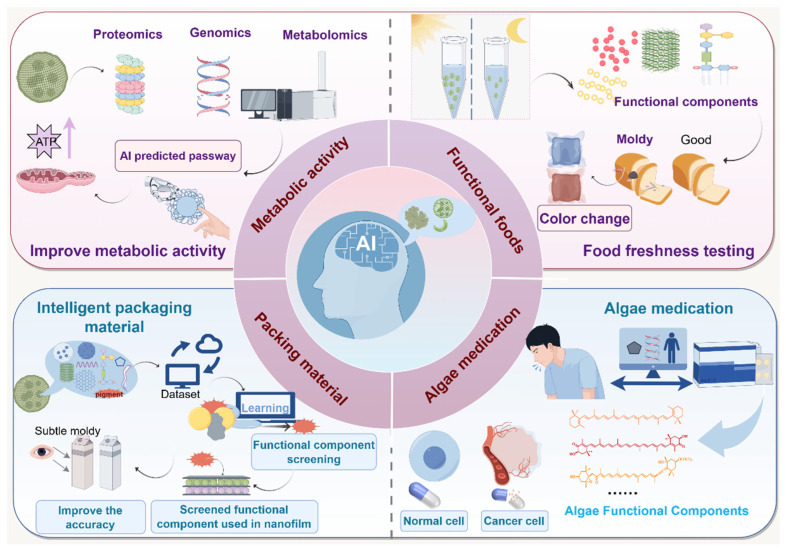
AI expands the application of algal bioactive compounds.

AI technology provides innovative support for developing personalized pharmaceuticals and nutrition products by deeply analyzing the biological characteristics and mechanisms of algal bioactive ingredients. Leveraging extensive biological databases and sophisticated ML models, AI establishes dynamic associations between algal metabolites and human health requirements, facilitating intelligent identification and synergistic research of functional components. This technology transcends traditional empirical research approaches, transitioning the application of algal bioactives from generalized mass production to precise, customized designs. Consequently, it significantly enhances development efficiency and maximizes potential value in functional foods, pharmaceuticals, and other related fields (Figure 3).

### 4.1. AI-Assisted Personalized Product Development Expands Algal Bioactive Application in Pharmaceuticals and Food

Algae offer diverse pharmaceutical products, proteins, vaccines, and nutritional supplements. Notable examples include astaxanthin from *Rhodococcus rainieri*, *Chlorella* as a dietary supplement, and β-carotene from *Dunaliella* [39]. Despite these established uses, many algal metabolites with bioactive properties remain unexplored. AI technology is thus crucial for broadening the application spectrum of algal bioactives in medical, nutritional, and agricultural sectors, thereby unlocking significant commercial and economic potential.

Integrating metabolomics, transcriptomics, and protein interaction network data, AI constructs dynamic multi-level metabolic pathway models, accurately delineating metabolic transformations of algal bioactives in biological systems. Graph neural network-based metabolic network reconstruction identifies critical catalytic nodes, elucidating molecular mechanisms, such as astaxanthin’s activation of antioxidant cascades via the Nrf2/ARE pathway [40]. Coupled with three-dimensional molecular docking and extensive conformational sampling, AI quantifies binding patterns of bioactives to target proteins, revealing conformational dynamics associated with anti-inflammatory effects [41]. A reinforcement learning-driven virtual physiological system combined with physiologically based pharmacokinetic (PB-PK) modeling simulates in vivo metabolic dynamics, successfully predicting bioavailability of compounds like fucoxanthin and associated metabolite toxicity. This digital twin approach significantly accelerates the research and development timeline for bioactive ingredients, notably reducing the development cycle by over 60% compared to traditional methods [42].

In functional food engineering, AI transforms photosensitive algal metabolites into innovative sensing technologies, extending their applications into emerging areas such as optical food freshness monitoring. For example, incorporating color-responsive metabolites like astaxanthin into functional food matrices enables the development of non-invasive intelligent sensing systems leveraging photosensitivity. DL-based dynamic shelf life prediction algorithms and transfer learning methods facilitate in situ optical diagnosis of food oxidation stability by correlating spectral data with oxidative indicators [43]. This approach utilizes hyperspectral imaging to monitor dynamic color changes, harnesses CNN modeling to establish relationships between color and oxidation, and employs transfer learning to adapt laboratory data for real-world storage conditions, enabling real-time, non-destructive freshness monitoring.

Remarkably, composite membranes combining algal pigments and polysaccharides can amplify color change signals dramatically through precise nanostructural modifications, detecting spoilage substances at ultra-low concentrations and visually indicating food spoilage through observable color transitions [44]. AI-driven ML systems optimize active preservation agents, such as algal polyphenols, within these composite membranes, simultaneously predicting medicinal efficacy and safety, thereby accelerating the development of functional packaging materials [12,45,46,47]. This synergy of AI and algal bioactive materials facilitates comprehensive resource utilization across the food preservation and pharmaceutical sectors, underpinning sustainable food–medical industry development.

### 4.2. AI Broadens the Innovative Applications of Algal Bioactive Ingredients

AI technology significantly expands the application potential of algal bioactive compounds across various high-value industries. In precision drug development, AI can engineer targeted algal-based therapeutics tailored to specific disease conditions. For instance, intelligent capsules designed using AI release drugs selectively within tumor-specific acidic environments, minimizing side effects [48]. Moreover, AI-enabled biomimetic designs, inspired by natural algal structures like diatom shells, could lead to self-healing building coatings capable of autonomously repairing structural cracks [49]. Furthermore, AI optimization of algal adsorption properties promises the development of marine cleanup particles that magnetically adsorb microplastics and degrade pollutants under sunlight, providing a sustainable, residue-free pollution control solution [50,51]. These technological breakthroughs transform algal bioactives from dietary supplements into valuable multifunctional materials with applications spanning pharmaceuticals, environmental protection, and construction.

## 5. Prospects

With increasing global awareness of health and environmental sustainability, the demand for natural and healthy food additives and functional foods is continually rising. Algae-derived bioactive ingredients are expected to experience substantial market growth due to their inherent health benefits and natural origin. AI, through intelligent systems and dynamic modeling, has significantly advanced the production, characterization, and application scope of algae bioactive ingredients. Despite these promising developments and cost reductions, numerous challenges remain.

### 5.1. Technical Challenges and AI-Driven Solutions in Algae Bioactive Ingredient Production

When applying AI to optimize algal cultivation conditions, several inherent constraints arise, notably contamination risks from competing bacteria or algae [52]. The complexity of algae biology and genetics represents another substantial barrier, particularly when developing microalgae as alternative protein sources. These processes involve numerous variables and require extensive, costly, and labor-intensive experiments. Challenges span multiple stages—from identifying safe algal strains and evaluating their protein production capabilities to assessing their adaptability to laboratory or bioreactor conditions [53,54]. In general, three primary technical challenges remain prominent. The first of these challenges is the limited databases: Growth characteristics vary significantly among algal species, yet most existing data pertain to commonly studied species. Rare algae with unique bioactive compounds lack sufficient data, complicating accurate predictions by AI models [12]. The second challenge is inadequate dynamic control: traditional AI models, typically trained on static datasets, fail to respond effectively to dynamic environmental fluctuations (e.g., temperature and light changes) [11]. The third challenge is interdisciplinary complexity: integrating knowledge across biology, chemistry, and engineering—spanning molecular-level genetic data to macro-level production equipment parameters—remains challenging for existing AI systems [3,4].

Specifically, AI-driven Industry 4.0 and ML models face issues concerning data quality, generalization, interpretability, and continual performance improvement. ML models heavily depend on the availability and accuracy of large datasets, often hindered by lengthy data collection processes and measurement errors inherent in industrial environments [12]. Additionally, inconsistent data intervals complicate dataset utilization. Moreover, time-series augmentation methods (e.g., time-warping, window slicing, interpolation) can address dataset insufficiencies. Zhang et al. introduced an innovative model combining transfer learning with transformer architecture, significantly enhancing predictive accuracy in limited datasets [55].

Future breakthroughs can focus on the following areas: (1) data acquisition, including the establishment of comprehensive “growth archives” covering diverse algal species through automated, real-time data collection; (2) algorithm development, encompassing the creation of adaptive AI models capable of responding autonomously to dynamic environmental changes; and (3) application expansion, involving the construction of virtual simulation platforms to digitally replicate the entire process—from genetic expression to industrial-scale cultivation—enabling virtual experiments prior to physical production. Additionally, establishing ecological risk assessment frameworks will be essential to prevent unintended environmental impacts.

### 5.2. Technical Challenges and AI-Driven Solutions in Characterization of Algae Bioactive Ingredients

AI currently faces critical bottlenecks in accurately tracking real-time dynamics and identifying trace bioactive components [1,12]. Traditional sampling methods fail to capture rapid fluctuations of metabolites under environmental stress (e.g., sudden changes in light intensity), and conventional spectral analysis often misses structurally similar bioactive molecules. To overcome these limitations, novel analytical strategies must be pursued. Integrating miniature spectroscopy sensors directly into cultivation monitoring systems can facilitate continuous tracking of metabolite dynamics, thereby constructing dynamic compositional maps [56]. Furthermore, developing AI models trained on comprehensive algal compound databases can enhance rapid structural inference of unknown components from minimal spectral data, significantly improving the accuracy and efficiency of component identification.

Although AI has significantly accelerated the molecular research and development of algal bioactive compounds, much of the planet’s algal diversity remains uncharacterized. Current taxonomy covers less than 10% of actual algal species, with rare or extremophilic algae in tropical, polar, and deep-sea ecosystems potentially producing novel metabolites of high pharmaceutical or industrial value. The conservation and exploration of these untapped molecular resources are vital for global sustainability and health innovation. AI enables efficient species discovery, functional prediction, and ecological assessment by integrating diverse datasets. More importantly, it supports a shift from extractive resource use to a smart, sustainable framework—aligning with the goals of the Convention on Biological Diversity and the United Nations’ Sustainable Development Goals (SDGs 14 and 15) and promoting biodiversity preservation through intelligent bioprospecting.

### 5.3. Technical Challenges and AI-Driven Solutions for Broadening Algal Ingredient Application

AI-driven synthetic biology strategies present transformative opportunities for engineering microalgae-based proteins. Deep neural networks can elucidate gene expression mechanisms in photosynthetic organisms, enabling the directed evolution of essential proteins such as ribulose diphosphate carboxylase. While engineered algal proteins hold potential as animal protein substitutes, consumer acceptance, particularly regarding sensory attributes, remains challenging. Existing metabolic reprogramming technologies struggle to accurately mimic complex meat flavors, and plant-derived proteins frequently lack certain essential amino acids [10]. Recent innovations in enzyme engineering, such as using generative adversarial networks (GANs) to design spatially selective proteases, demonstrate significant promise. These methods enable controlled protein cleavage, generating bioactive peptide profiles similar to gastrointestinal digestion products. Such biomimetic strategies retain essential amino acids and regulate sensory properties, addressing consumer acceptance hurdles [57,58]. Coupled with flavor adaptation algorithms driven by metabolomics, these advances pave the way for a new generation of intelligent, plant-based protein products. Moreover, AI-powered intelligent packaging utilizing algae-derived color indicators also offers a breakthrough solution for food preservation monitoring. For example, astaxanthin-based composite films visibly respond to microbial growth, allowing for real-time quality monitoring [59]. By integrating these films with machine vision technology, predictive models linking color changes to shelf life can significantly enhance supply chain transparency and product safety [44].

To facilitate broader industrial application of algae bioactive ingredients, critical technical barriers—particularly multi-source data integration, AI model generalization, interpretability, and continual performance enhancement—must be addressed. Leveraging AI innovations, algae-based products can increasingly serve as sustainable protein alternatives, effectively promoting wider personalized applications in food, healthcare, and beyond, thus advancing food sustainability and the circular economy.

### 5.4. The Regulation of AI in Algae Research

As AI becomes central to algal research, ethical and regulatory concerns must not be overlooked. These include fair access to genetic resources, protection of traditional knowledge, and data sovereignty—especially for megadiverse countries that hold much of the world’s biodiversity. Without safeguards, AI could deepen inequities between technologically advanced nations and biodiversity-rich regions, raising concerns over data sovereignty, benefit sharing, and environmental justice. Therefore, future development must incorporate transparent regulatory frameworks, promote fair technological transfer, and ensure that AI-driven innovations align with international agreements such as the Nagoya Protocol on access and benefit sharing. Ethical foresight and inclusive governance are essential to ensure that AI enhances autonomy and supports global biodiversity justice.

## 6. Conclusions

AI has provided revolutionary momentum to the development of functional algal ingredients, fundamentally transforming traditional research methodologies and significantly accelerating industrial translation efficiency. Algae produce a wide array of high-value bioactive compounds, which are widely utilized in the food, pharmaceutical, and cosmetics industries. However, conventional development strategies remain constrained by limited production efficiency, insufficient characterization of active components, and restricted application scope. The integration of AI addresses these limitations through three core strategies. Firstly, AI enables the real-time control of cultivation processes and regulation of encoding genes, significantly boosting the productivity of targeted bioactives. Secondly, AI-driven high-throughput spectral imaging and recognition technologies facilitate rapid and precise identification and quality assessment of bioactive compounds. Thirdly, AI expands the innovative applications of algal bioactive compounds beyond traditional sectors, unlocking potential in novel industries. Collectively, these advancements signify a pivotal transition towards personalized, sustainable, and intelligent green manufacturing within the algae industry. Future technological breakthroughs should prioritize the establishment of biologically interpretable AI models and universal frameworks for metabolic knowledge transfer. Ultimately, AI is poised to become an indispensable force driving the advancement and broader utilization of algal bioactive compounds.

## Data Availability

No new data were created or analyzed in this study. Data sharing is not applicable to this article.
